# Total flavonoids of Radix Tetrastigma suppress inflammation-related hepatocellular carcinoma cell metastasis

**DOI:** 10.1007/s00438-020-01759-6

**Published:** 2021-02-12

**Authors:** Zhendong Liu, Fangmi Ding, Xingyong Shen

**Affiliations:** 1 The First Affiliated Hospital of Zhejiang Chinese Medical University (Zhejiang Provincial Hospital of TCM), Hangzhou, 310006 China; 2 Department of Oncology, Xijing Hospital, Air Force Military Medical University, 15 Changle West Road, Xian, 710032 Shaanxi China

**Keywords:** Radix Tetrastigma, TLR4, NF-κB/p65, Hepatocellular carcinoma

## Abstract

This study aimed to investigate the effects of the total flavonoids of Radix Tetrastigma (RTF) on inflammation-related hepatocellular carcinoma (HCC) development. Extracted RTF was diluted to different concentrations for subsequent experiments. HCC cells were cotreated with lipopolysaccharide (LPS) and RTF to investigate the effects of RTF on LPS-stimulated HCC cells. A CCK-8 kit was used to measure cell proliferation. Apoptosis was detected with a flow cytometer. Cell migration and invasion were quantified by wound healing and Transwell assays, respectively. The expression of TLR4 and COX-2 and activation of the NF-κB pathway were determined by Western blotting. Treatment with LPS significantly enhanced cell proliferation and decreased the apoptosis rate, while cell migration and invasion were notably upregulated. RTF suppressed the proliferation and invasion induced by LPS stimulation and promoted HCC cell apoptosis. The protein levels of Bax and cleaved caspase-3 were decreased and that of Bcl-2 was increased by LPS in HCC cells, which could be rescued by RTF. RTF significantly inhibited the LPS-induced expression of the proinflammatory mediators IL-6 and IL-8 in HCC cells. Mechanistically, with RTF treatment, the upregulated expression of TLR4 and COX-2 induced by LPS was obviously downregulated. Furthermore, the phosphorylation of NF-κB/p65 was significantly decreased in LPS-stimulated cells after supplementation with RTF. Our study suggests that RTF exerts a significant inhibitory effect on the LPS-induced enhancement of the malignant behaviors of HCC cells via inactivation of TLR4/NF-κB signaling. RTF may be a promising chemotherapeutic agent to limit HCC development and inflammation-mediated metastasis.

## Introduction

Hepatocellular carcinoma (HCC) is a common cancer with high degrees of malignancy, infiltration, and metastasis (Dhanasekaran et al. 2012; Ou et al. 2018). There are approximately 1 million new cases of liver cancer diagnosed in the world every year, and the annual death rate exceeds 700,000, with the proportion in China accounting for more than two-fifths of this total (Forner et al. 2012; Bruix et al. 2014). Currently, there is no specific treatment for HCC. Surgical resection is considered to be the most effective method for the treatment of early-stage primary liver cancer, but most liver cancers are in advanced stages when diagnosed in the clinic. Surgical treatment alone has not been satisfactory, and a large percentage of patients remain at great risk for recurrence and metastasis after surgery (Mazzola et al. 2015; Palmer and Johnson 2015; Zhang et al. 2017). A large number of studies have proven that interventional therapy can prolong the survival time of liver cancer patients and allow some patients to regain the opportunity for surgical resection (Aguayo and Patt 2001; Kristiansen et al. 2002; Jiang et al. 2017). However, the traditional chemotherapeutic drugs used in interventional therapy have high toxicity and notable side effects, which may cause further liver damage and even liver failure. Therefore, developing specific drugs with low toxicity and few or mild side effects for HCC treatment has become an urgent task facing China and the world today.

At present, a large number of studies have proven that the lipopolysaccharide (LPS)/Toll-like receptor 4 (TLR4) signaling pathway plays an important role in chronic inflammatory diseases and that chronic inflammation is closely related to tumor development (Li et al. 2017, 2019). Chronic inflammation can create a favorable tumor microenvironment conducive to tumor growth and metastasis. In a study of the liver cancer microenvironment, an important feature of the liver tumor microenvironment was found to be the persistence of bacterial antigen components, such as LPS (Dapito et al. 2012; Gu et al. 2015). As an LPS receptor, TLR4 cannot only regulate inflammatory responses but also affect noninfectious inflammatory conditions, such as tumor invasion (Seifert et al. 2015). Studies have found that silencing TLR4 expression can inhibit invasion in prostate cancer, suggesting that TLR4 plays an important role in tumor invasion. In addition, there are some studies showing that LPS can promote liver cancer cell invasion through TLR4/NF-κB signaling (Dapito et al. 2012). Therefore, LPS/TLR4 signaling may also be a potentially important target for the treatment of HCC.

Radix Tetrastigma (RTF, *Tetrastigma hemsleyanum Diels et Gilg*) contains many pharmacological compounds, such as quercetin, kaempferol, kaempferol-3-*O*-neohesperidin, and other flavonoids (Peng et al. 2016a). In traditional Chinese medicine, RTF is often used for the treatment of infantile convulsion, pneumonia, bronchitis, sore throat, dysentery, hepatitis, and viral meningitis. In addition, RTF is commonly used in tumor treatment (Feng et al. 2014; Xiong et al. 2015; Lin et al. 2016b). For liver cancer, a previous study reported that ethylacetate extracted from RTF has strong inhibitory effects on the activity of HepG2 human liver cancer cells (Peng et al. 2016b). Similarly, quercetin, one of the compounds that can be extracted from RTF, was found to have significant antiproliferative and apoptosis-inducing effects on a human colon cancer cell line (Xu et al. 2015). Furthermore, other studies have suggested that treatment with RTF can induce loss of the mitochondrial membrane potential and promote the activation of caspase-8, which could lead to human cervical cancer cell death (Xiong et al. 2015). Based on this evidence, this study focused on the effects of RTF on the LPS-induced proliferation, migration, and invasion of HCC cells.

In this study, LPS-stimulated HCC cells were treated with different concentrations of the total flavones of RTF and monitored to detect changes in various biological behaviors, such as cell proliferation and migration. Then, to explore the potential molecular mechanism of the regulation of HCC progression by the total flavones of RTF, the activation of the TLR4/Cyclooxygenase-2 (COX-2) pathway in each group of cells was detected by Western blotting.

## Materials and methods

### Extraction of Radix Tetrastigma

Five kilograms of RTF was used for the extraction by hot reflux extraction with 60% ethanol. The extraction was performed 3 times for 1.5 h each time. The filtrates were combined and concentrated under reduced pressure to obtain an extract. The extract was added to an HPD826 macroporous adsorption resin column and washed with water and different concentrations of ethanol, followed by collection of the corresponding eluent. Then, the ethanol phase was recovered, concentrated into a thick paste, and dried in vacuo. Finally, the total flavones from RTF were obtained. All analyses of the extracts were performed using a high-performance liquid chromatography tandem mass spectrometry (HPLCMS/MS) method.

### Cell culture

HCC cell lines (Huh7 and HepG2) were purchased from ATCC (Manassas, VA, USA). All cells were cultured in RPMI 1640 medium (Gibco, Carlsbad, CA, USA) supplemented with 50 U/ml streptomycin, 50 U/ml penicillin, and 10% fetal bovine serum (Gibco, Carlsbad, CA, USA) in 5% CO_2_ at 37 °C. To investigate the effects of RTF on LPS-induced HCC cells, cells were cotreated with 1 μg/ml LPS and different concentrations of RTF (5 and 10 mg/ml) for 48 h. RTF at a concentration of 5 mg/ml was defined as RTF-L, and RTF at a concentration of 10 mg/ml was defined as RTF-H. Then, the cells were collected to evaluate the anticancer effects of RTF.

### CCK-8 assay

After cells were cotreated with LPS and RTF-L (5 mg/ml) or RTF-H (10 mg/ml), the growth inhibition rate of each group of cells was measured by a CCK-8 assay. First, treated cells were added to CCK-8 reagent (MedChemExpress (MCE), Shanghai, China) and incubated for 4 h in a 5% CO_2_ incubator at 37 °C. The absorbance at 450 nm was measured by a microplate reader to calculate the cell survival rate. Three replicates of each group of cells were measured in parallel.

### Flow cytometric analysis of apoptotic cell death

Flow cytometry was applied to evaluate cell apoptosis with the Annexin V-conjugated Alexa Fluor 488 (Alexa488) Apoptosis Detection Kit (Invitrogen) following the instructions of the manufacturer. In brief, after cotreatment with LPS and RTF, cells were collected and incubated with Alexa488 and propidium iodide (PI). The stained cells were analyzed by fluorescence-activated cell sorting (FACS) using a FACSCalibur instrument (BD Biosciences) equipped with Cell Quest 3.3 software.

### Wound healing assay

Collected cells were added to a 6-well culture dish (5 × 10^5^ cells/well) overnight. The next day, a pipette tip was used to create a wound across the confluent cell monolayer, followed by incubation in 5% CO_2_ at 37 °C. Photos of the wound were captured at 0 and 24 h to calculate cell migration.

### Transwell assay

Collected cells were placed in the upper chamber of 24-well Transwell chambers (8-μm pore size) filled with FBS-free medium. The lower chamber was filled with medium containing 10% FBS. After 24 h, the Matrigel and cells in the upper side of the membrane were removed with a cotton swab, and the cells in the lower chambers were fixed in 4% paraformaldehyde for 10 min and stained with a 1% crystal violet staining solution. At least 5 representative pictures of each well were captured, and cell numbers were counted using ImageJ.

### Enzyme-linked immunosorbent assay (ELISA)

The IL-6 and IL-8 levels in cell culture medium were determined with enzyme-linked immunosorbent assay (ELISA) kits (R&D Systems, Minneapolis, MN) according to the manufacturer’s instructions. Each measurement was repeated in triplicate.

### Western Blotting

All treated cells were lysed, and total protein was extracted. The lysates were collected and centrifuged at 4 °C and 12,000×*g* for 20 min to pellet cell debris. A BCA protein assay (Solarbio, Beijing, China) was applied to quantify the concentration of extracted protein. The total protein isolated from treated HCC cells was subjected to 10% sodium dodecyl sulfate–polyacrylamide gel electrophoresis. Then, the proteins were transferred to a PVDF membrane (GE Healthcare Life, Beijing, China), followed by blocking with 5% nonfat milk. After that, the membrane was incubated with a primary antibody overnight at 4 °C. After incubation with the corresponding secondary antibodies, the membrane was washed with TBST buffer, and target proteins were detected with enhanced chemiluminescence (ECL) reagents (Beyotime, Shanghai, China). All primary antibodies are listed in Table 1.

**Table1:** Primary antibodies

Primary antibody	Business	Number
Anti-Bax antibody	Abcam	(ab32503)
Anti-Bcl-2 antibody	Abcam	(ab692)
Anti-cleaved caspase-3 antibody	Abcam	(ab49822)
Anti-TLR4 antibody	Abcam	(ab22048)
Cox2 antibody	CST	4842S
Phospho-NF-κB p65 (Ser536) (93H1) Rabbit mAb	CST	3033S
NF-κB p65 (D14E12)XP® Rabbit mAb	CST	8242S

### Statistics

Statistical differences between the respective controls and each experimental test condition were determined by one-way analysis of variance (ANOVA), followed by Bonferroni test. *p* < 0.01 and *p* < 0.05 were considered significant. Values are expressed as the mean ± SD. Each experiment was repeated at least three times.

## Results

### RTF shows obvious inhibitory effects on the LPS-induced increases in HCC cell proliferation and survival

**Fig. 1:**
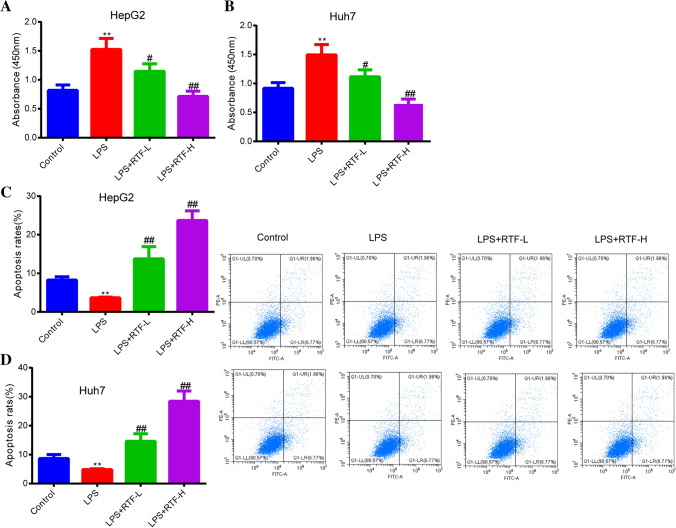
RTF showing obvious inhibitory effects on the LPS-induced increases in HCC cell proliferation and survival. **a**, **b** The effects of RTF (5 and 10 mg/ml) on the elevated HepG2 and Huh7 cell proliferation induced by LPS were measured by a CCK-8 assay. **c**, **d** Changes in the apoptosis of HepG2 and Huh7 cells treated with LPS and RTF (5 and 10 mg/ml) were detected by flow cytometry. *p < 0.05 and **p < 0.01 vs. the control group; ^#^*p* < 0.05 and ^##^*p* < 0.01 vs. the LPS group. Each experiment was repeated at least three times

To investigate the effects of RTF on the LPS-induced increase in cell proliferation, two types of HCC cells were subjected to treatment with LPS and RTF. As shown in Fig. 1a, b, LPS treatment alone significantly promoted the proliferation of both HepG2 cells and Huh7 cells (*p* < 0.01). When cells were treated with LPS and RTF together, cell proliferation was dramatically decreased in the LPS + RTF-L/H groups compared to the LPS groups (LPS + RTF-L, *p* < 0.05; LPS + RTF-H, *p* < 0.01). The inhibitory effects of RTF on cell proliferation were dose dependent. Furthermore, flow cytometry was used to quantify cell apoptosis. The results shown in Fig. 1c, d indicate that LPS treatment could further reduce apoptosis in HepG2 and Huh7 cells (*p* < 0.01). However, supplementation with RTF led to an impressive increase in the apoptosis rates (*p* < 0.01). Overall, RTF shows obvious inhibitory effects on the LPS-induced increases in the cell proliferation and survival of HCC cells.

### RTF suppresses the enhanced migratory and invasive abilities induced by LPS in HCC cells

**Fig. 2:**
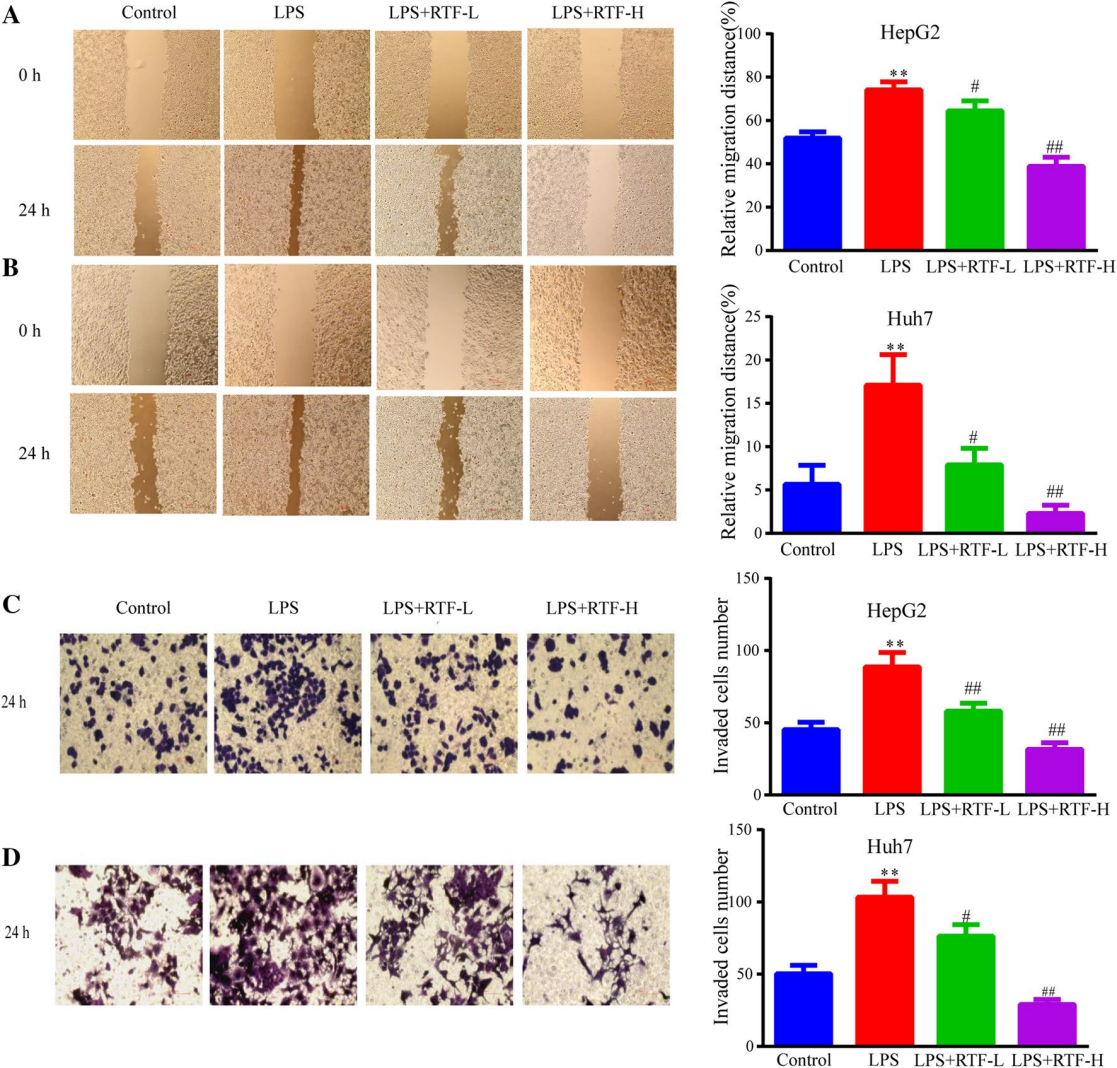
RTF suppressing the enhanced migratory and invasive abilities induced by LPS in HCC cells. **a**, **b** The effects of RTF (5 and 10 mg/ml) on the elevated migration rate of HepG2 and Huh7 cells induced by LPS treatment were evaluated by a wound healing assay. **c**, **d** Changes in the invaded HepG2 and Huh7 cells in response to treatment with LPS and RTF (5 and 10 mg/ml) were measured by a Transwell assay. *p < 0.05 and **p < 0.01 vs. the control group; ^#^*p* < 0.05 and ^##^*p* < 0.01 vs. the LPS group. Each experiment was repeated at least three times

We also measured changes in the cell migration and invasion of HepG2 and Huh7 cells under treatment with LPS and RTF. Similarly, both the migratory ability and invasive ability of HCC cells were significantly elevated in the LPS group (*p* < 0.01, Fig. 2a–d). In comparison to the LPS groups, the LPS + RTF-L/H groups showed much decreased migration and invasion for both HepG2 and Huh7 cells (LPS + RTF-L, *p* < 0.05; LPS + RTF-H, *p* < 0.01, Fig. 2a–d). Therefore, RTF significantly suppresses not only the LPS-induced increase in HCC cell proliferation but also the induced increases in migration and invasion.

### RTF negatively regulates apoptotic protein expression and inflammatory cytokine generation in LPS-stimulated HCC cells

**Fig. 3:**
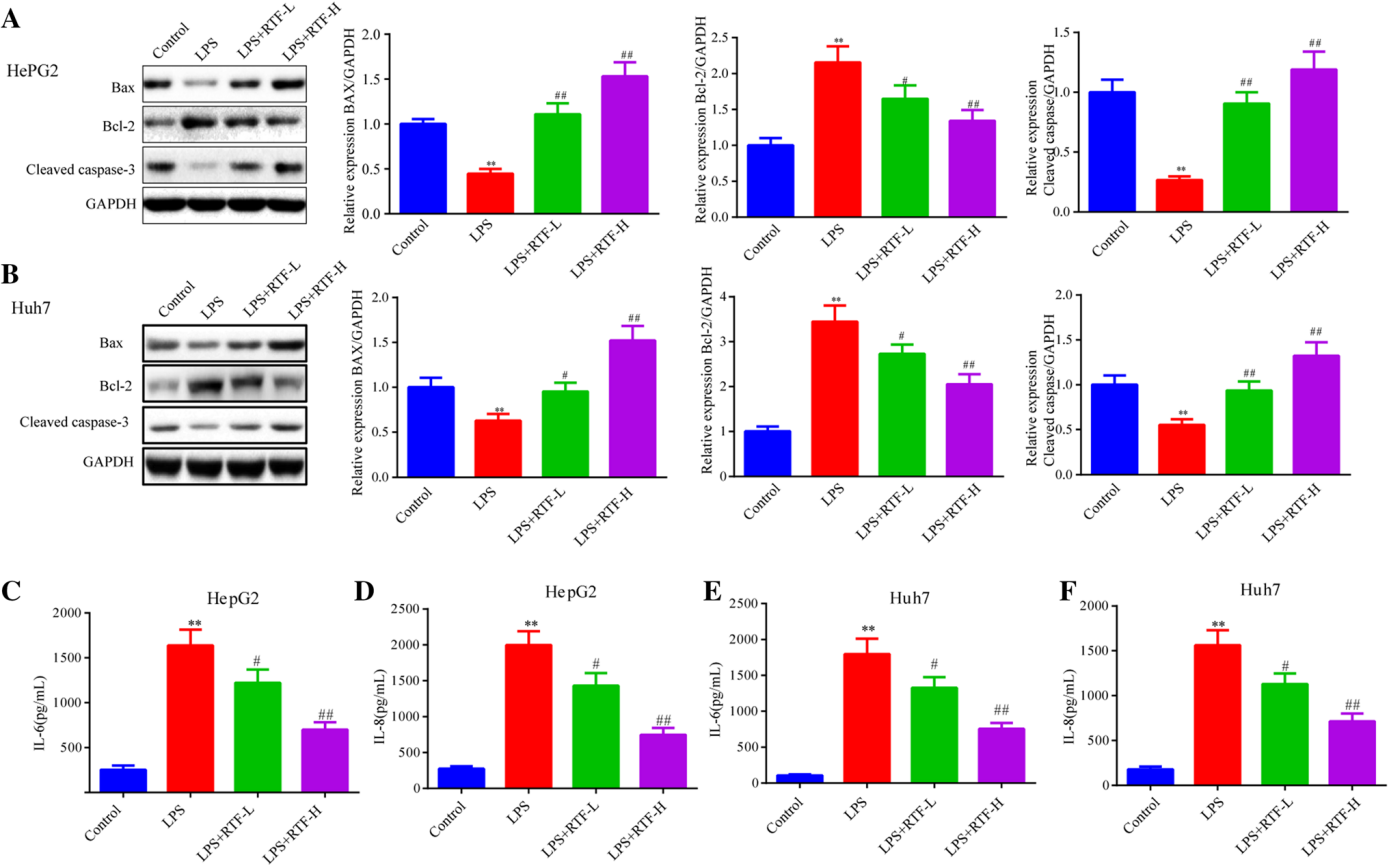
RTF negatively regulating apoptotic protein expression and inflammatory cytokine generation in LPS-stimulated HCC cells. **a**, **b** We detected the protein levels of Bcl-2, Bax, and cleaved caspase-3 in HepG2 and Huh7 cells by Western blotting. GAPDH served as an internal control. **c**–**f** The concentrations of the inflammatory cytokines IL-6 and IL-8 were quantified with commercially available ELISA kits. *p < 0.05 and **p < 0.01 vs. the control group; ^#^*p* < 0.05 and ^##^*p* < 0.01 vs. the LPS group. Each experiment was repeated at least three times

First, we detected the expression of apoptosis-related proteins in HepG2 and Huh7 cells treated with LPS and RTF. As shown in Fig. 3a, b, after treatment with LPS alone, the protein levels of Bcl-2 in HepG2 and Huh7 cells were significantly upregulated, and the protein levels of Bax and cleaved caspase-3 were obviously decreased (*p* < 0.01). Compared with this, RTF treatment showed a strong suppressive effect on Bcl-2 protein levels, and the protein levels of Bax and cleaved caspase-3 were markedly upregulated. Then, we also measured the levels of inflammatory cytokines (IL-6 and IL-8) by ELISA. After LPS treatment, the levels of IL-6 and IL-8 in the cell supernatant were significantly increased compared to after control treatment (*p* < 0.01, Fig. 3c–f). When cells were subjected to cotreatment with LPS and RTF, the levels of IL-6 and IL-8 in the cell supernatant showed obvious decreases (LPS + RTF-L, *p* < 0.05; LPS + RTF-H, *p* < 0.01, Fig. 3c–f). Therefore, these results suggested that RTF enhanced the apoptosis rates of HCC cells by regulating the expression of apoptosis-related proteins. RTF also showed a promising effect on the proinflammatory response induced by LPS in HCC cells.

### RTF suppresses the activation of NF-κB/p65 in LPS-stimulated HCC cells

**Fig. 4:**
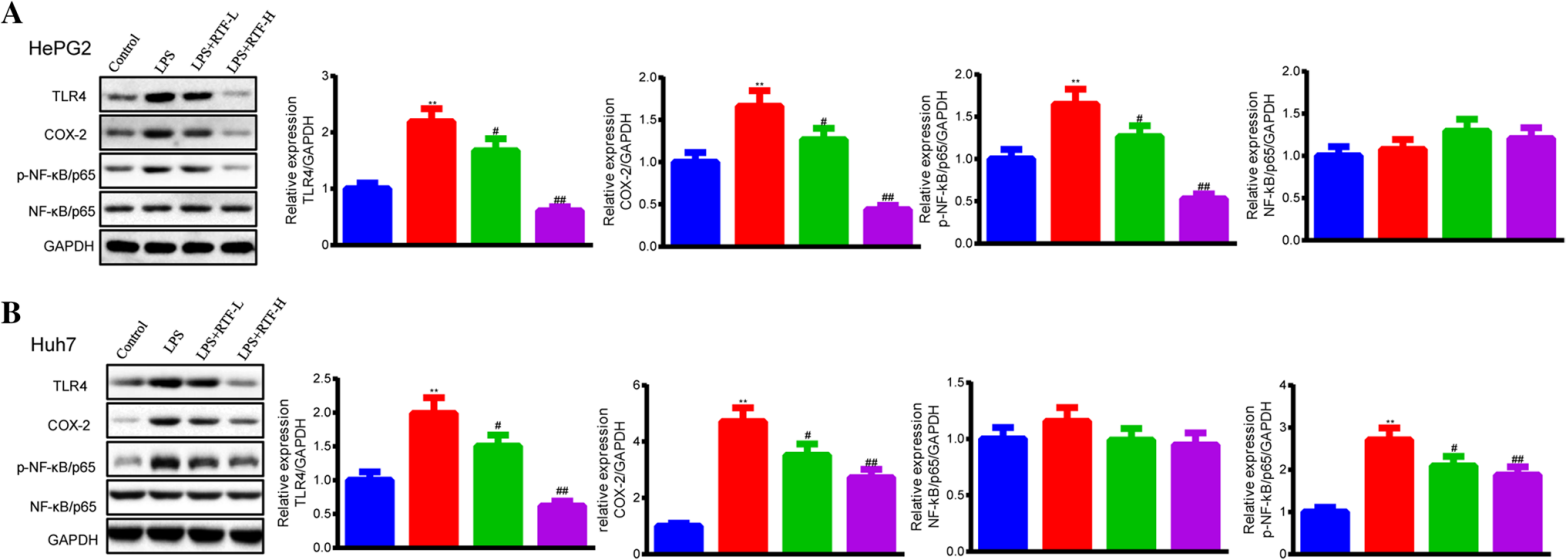
RTF suppressing the activation of NF-κB/p65 in LPS-stimulated HCC cells. **a** We detected the protein levels of TLR4, COX-2, p-NF-κB/p65, and NF-κB/p65 in HepG2 cells treated with LPS and RTF by Western blotting. **b** We detected the protein levels of TLR4, COX-2, p-NF-κB/p65, and NF-κB/p65 in Huh7 cells treated with LPS and RTF by Western blotting. GAPDH served as an internal control. *p < 0.05 and **p < 0.01 vs. the control group; ^#^*p* < 0.05 and ^##^*p* < 0.01 vs. the LPS group. Each experiment was repeated at least three times

To further investigate the mechanism underlying the effects of RTF on the LPS-induced enhancement of malignant behaviors in HCC cells, we detected the expression of TLR4 and the downstream gene COX-2_,_ and the activation of the NF-κB signaling pathway. We first measured the protein levels of TLR4 and COX-2. As shown in Fig. 4a, b, LPS treatment significantly upregulated the protein levels of TLR4 and COX-2 compared to control treatment (*p* < 0.01), while RTF supplementation obviously abolished the LPS-induced upregulation of TLR4 and COX-2 expression (LPS + RTF-L, *p* < 0.05; LPS + RTF-H, *p* < 0.01). Then, we further evaluated the activation of the NF-κB pathway based on the level of phosphorylated NF-κB/p65. In comparison to the control groups, the LPS groups showed significantly increased levels of p-NF-κB/p65 in HepG2 and Huh7 cells (*p* < 0.01, Fig. 4a, b); in contrast, the levels of phosphorylated NF-κB/p65 in the LPS + RTF groups were notably decreased, but there was no difference in the protein levels of NF-κB/p65.

## Discussion

In this study, we focused on the effects of RTF on the increases in HCC cell survival, migration, and invasion induced by LPS. First, we demonstrated that RTF treatment could obviously suppress the increased proliferation of Huh7 and HepG2 cells induced by LPS, and the results of wound healing and Transwell assays suggested that RTF had the ability to attenuate the migration and invasion of HCC cells induced by LPS stimulation. To further investigate the mechanism underlying the effects of RTF on LPS-stimulated HCC cells, we also detected the expression of the typical LPS receptor TLR4 and the activation of the NF-κB pathway. Overall, RTF treatment significantly inhibited cell proliferation, migration, and invasion in LPS-stimulated HCC cells, and inactivation of the TLR4/NF-κB pathway may play an important role in the antitumor effects of RTF.

Increasing evidence suggests that chronic inflammation plays an important role in the development and metastatic progression of cancer (Liu et al. 2017). RTX contains numerous compounds, and many of them have been proven to have anti-inflammatory, antioxidative, and antitumor properties (Wang and Jang 2018; Chu et al. 2019; Ji et al. 2019). Quercetin is one of the flavonoids extracted from RTX, and numerous studies have proven that quercetin treatment notably inhibits the expression of TLR4 and activation of the NF-κB signaling pathway (Junyuan et al. 2018; Wu et al. 2018b). Additionally, kaempferol was also found to have strong inhibitory effects on inflammatory cytokines through inactivation of the NF-κB pathway mediated by downregulating the expression of TLR4 (Cheng et al. 2018; Zhong et al. 2018). Considering these findings, we speculated that the anti-inflammatory effects of RTX may also be able to suppress the development and metastatic progression of HCC cells.

There have been numerous studies on the promotive effects of TLR4 expression on the development and metastatic progression of different cancer types (Pandey et al. 2018; Ren et al. 2018; Wu et al. 2018a). In 2018, Wu et al. found that the activation of TLR4/myeloid differentiation factor (MyD) 88 signaling significantly enhanced the migratory and invasive abilities of breast cancer cells and that the expression of TLR4/MyD88 was significantly positively correlated with breast cancer cell metastasis (Wu et al. 2018a). In addition, Zhou et al. proved that Galectin-3 could obviously promote the proliferation of lung adenocarcinoma cells by activating TLR4/NF-κB signaling (Zhou et al. 2018). Collectively, these studies suggest promotive roles for TLR4 in the development and metastasis of cancer. Here, we also found that LPS treatment could significantly promote the expression of TLR4 and activation of NF-κB signaling, which, in turn, enhanced the cell survival rate, migration, and invasion. Another study proved that TLR4 and COX-2 are highly expressed in prostate cancer cells and showed that the inhibition of TLR4 and COX-2 obviously suppressed cell proliferation, migration, and invasion (Wang and Wang 2018). TAK-242, a specific inhibitor of TLR4, was reported to considerably attenuate epithelial–mesenchymal transition (EMT) in ovarian and breast cancer cells by suppressing extracellular degradation through targeting TLR4 and regulating matrix metalloproteinase-2 (MMP-2) and MMP-9 expression (Zandi et al. 2019). More importantly, Zhou et al. proved that TLR4 expression was substantially upregulated in tumor tissues from HCC patients compared to adjacent tissues (Yao et al. 2018). In addition, other research has demonstrated the promotive role for TLR4 in the development of HCC cells, and TLR4 inhibition was shown to have strong effects on LPS-induced inflammation and HCC cell proliferation (Lin et al. 2016a; Zhou et al. 2019). Similar to these studies, the results in our studies suggested that RTF could significantly inhibit the expression of TLR4, as well as the activation of the NF-κB pathway, in HCC cells, which resulted in the suppression of various malignant behaviors of HCC cells.

To conclude, this study demonstrated the anticancer effects of RTF on the increased cell proliferation and the enhanced migratory and invasive abilities of HCC cells induced by LPS. Furthermore, our results also showed that the expression of TLR4 and activation of NF-κB signaling were notably suppressed with RTF treatment. Taken together, these findings suggest that RTF should be further explored as a promising chemotherapeutic agent to limit HCC development and inflammation-mediated metastasis.

## Data Availability

The data analyzed during the current study are available from the corresponding author on reasonable request.

## References

[CR1] Aguayo A, Patt YZ (2001). Nonsurgical treatment of hepatocellular carcinoma. Semin Oncol.

[CR2] Bruix J, Gores GJ, Mazzaferro V (2014). Hepatocellular carcinoma: clinical frontiers and perspectives. Gut.

[CR3] Cheng X, Yang YL, Yang H, Wang YH, Du GH (2018). Kaempferol alleviates LPS-induced neuroinflammation and BBB dysfunction in mice via inhibiting HMGB1 release and down-regulating TLR4/MyD88 pathway. Int Immunopharmacol.

[CR4] Chu Q, Jia R, Chen W, Liu Y, Li Y, Ye X, Jiang Y, Zheng X (2019). Purified Tetrastigma hemsleyanum vines polysaccharide attenuates EC-induced toxicity in Caco-2 cells and *Caenorhabditis elegans* via DAF-16/FOXO pathway. Int J Biol Macromol.

[CR5] Dapito DH, Mencin A, Gwak GY, Pradere JP, Jang MK, Mederacke I, Caviglia JM, Khiabanian H, Adeyemi A, Bataller R, Lefkowitch JH, Bower M, Friedman R, Sartor RB, Rabadan R, Schwabe RF (2012). Promotion of hepatocellular carcinoma by the intestinal microbiota and TLR4. Cancer Cell.

[CR6] Dhanasekaran R, Limaye A, Cabrera R (2012). Hepatocellular carcinoma: current trends in worldwide epidemiology, risk factors, diagnosis, and therapeutics. Hepat Med.

[CR7] Feng Z, Hao W, Lin X, Fan D, Zhou J (2014). Antitumor activity of total flavonoids from Tetrastigma hemsleyanum Diels et Gilg is associated with the inhibition of regulatory T cells in mice. Onco Targets Ther.

[CR8] Forner A, Llovet JM, Bruix J (2012). Hepatocellular carcinoma. Lancet.

[CR9] Gu J, Sun R, Shen S, Yu Z (2015). The influence of TLR4 agonist lipopolysaccharides on hepatocellular carcinoma cells and the feasibility of its application in treating liver cancer. Onco Targets Ther.

[CR10] Ji W, Peng X, Lou T, Wang J, Qiu W (2019). Total flavonoids from Tetrastigma hemsleyanum ameliorates inflammatory stress in concanavalin A-induced autoimmune hepatitis mice by regulating Treg/Th17 immune homeostasis. Inflammopharmacology.

[CR11] Jiang C, Long J, Liu B, Xu M, Wang W, Xie X, Wang X, Kuang M (2017). miR-500a-3p promotes cancer stem cells properties via STAT3 pathway in human hepatocellular carcinoma. J Exp Clin Cancer Res.

[CR12] Junyuan Z, Hui X, Chunlan H, Junjie F, Qixiang M, Yingying L, Lihong L, Xingpeng W, Yue Z (2018). Quercetin protects against intestinal barrier disruption and inflammation in acute necrotizing pancreatitis through TLR4/MyD88/p38MAPK and ERS inhibition. Pancreatology.

[CR13] Kristiansen G, Denkert C, Schluns K, Dahl E, Pilarsky C, Hauptmann S (2002). CD24 is expressed in ovarian cancer and is a new independent prognostic marker of patient survival. Am J Pathol.

[CR14] Li J, Yin J, Shen W, Gao R, Liu Y, Chen Y, Li X, Liu C, Xiang R, Luo N (2017). TLR4 promotes breast cancer metastasis via Akt/GSK3beta/beta-catenin pathway upon LPS stimulation. Anat Rec.

[CR15] Li N, Xu H, Ou Y, Feng Z, Zhang Q, Zhu Q, Cai Z (2019). LPS-induced CXCR7 expression promotes gastric cancer proliferation and migration via the TLR4/MD-2 pathway. Diagn Pathol.

[CR16] Lin A, Wang G, Zhao H, Zhang Y, Han Q, Zhang C, Tian Z, Zhang J (2016). TLR4 signaling promotes a COX-2/PGE2/STAT3 positive feedback loop in hepatocellular carcinoma (HCC) cells. Oncoimmunology.

[CR17] Lin Z, Chen L, Qiu Q, Guo S (2016). Isolation and identification of antiproliferative compounds from the roots of Tetrastigma hemsleyanum against MDA-MB-435S cell lines. Pak J Pharm Sci.

[CR18] Liu X, Zhao W, Wang W, Lin S, Yang L (2017). Puerarin suppresses LPS-induced breast cancer cell migration, invasion and adhesion by blockage NF-kappaB and Erk pathway. Biomed Pharmacother.

[CR19] Mazzola A, Costantino A, Petta S, Bartolotta TV, Raineri M, Sacco R, Brancatelli G, Camma C, Cabibbo G (2015). Recurrence of hepatocellular carcinoma after liver transplantation: an update. Future Oncol.

[CR20] Ou Y, He J, Liu Y (2018). MiR-490-3p inhibits autophagy via targeting ATG7 in hepatocellular carcinoma. IUBMB Life.

[CR21] Palmer DH, Johnson PJ (2015). Evaluating the role of treatment-related toxicities in the challenges facing targeted therapies for advanced hepatocellular carcinoma. Cancer Metastasis Rev.

[CR22] Pandey N, Chauhan A, Jain N (2018). TLR4 polymorphisms and expression in solid cancers. Mol Diagn Ther.

[CR23] Peng X, Wu X, Ji Q, Yang R, Li Y (2016). Molecular authentication of Tetrastigma hemsleyanum from its adulterant species using ISSR, CAPS, and ITS2 barcode. Mol Biol Rep.

[CR24] Peng X, Zhang YY, Wang J, Ji Q (2016). Ethylacetate extract from Tetrastigma hemsleyanum induces apoptosis via the mitochondrial caspase-dependent intrinsic pathway in HepG2 cells. Tumour Biol.

[CR25] Ren B, Luo S, Tian X, Jiang Z, Zou G, Xu F, Yin T, Huang Y, Liu J (2018). Curcumin inhibits liver cancer by inhibiting DAMP molecule HSP70 and TLR4 signaling. Oncol Rep.

[CR26] Seifert L, Deutsch M, Alothman S, Alqunaibit D, Werba G, Pansari M, Pergamo M, Ochi A, Torres-Hernandez A, Levie E, Tippens D, Greco SH, Tiwari S, Ly NNG, Eisenthal A, van Heerden E, Avanzi A, Barilla R, Zambirinis CP, Rendon M, Daley D, Pachter HL, Hajdu C, Miller G (2015). Dectin-1 regulates hepatic fibrosis and hepatocarcinogenesis by suppressing TLR4 signaling pathways. Cell Rep.

[CR27] Wang CY, Jang HJ (2018). Alkaloids from tetrastigma hemsleyanum and their anti-inflammatory effects on LPS-induced RAW264.7 cells. Molecules.

[CR28] Wang W, Wang J (2018). Toll-like receptor 4 (TLR4)/cyclooxygenase-2 (COX-2) regulates prostate cancer cell proliferation, migration, and invasion by NF-kappaB activation. Med Sci Monit.

[CR29] Wu K, Zhang H, Fu Y, Zhu Y, Kong L, Chen L, Zhao F, Yu L, Chen X (2018). TLR4/MyD88 signaling determines the metastatic potential of breast cancer cells. Mol Med Rep.

[CR30] Wu TC, Chan ST, Chang CN, Yu PS, Chuang CH, Yeh SL (2018). Quercetin and chrysin inhibit nickel-induced invasion and migration by downregulation of TLR4/NF-kappaB signaling in A549 cells. Chem Biol Interact.

[CR31] Xiong Y, Wu X, Rao L (2015). Tetrastigma hemsleyanum (Sanyeqing) root tuber extracts induces apoptosis in human cervical carcinoma HeLa cells. J Ethnopharmacol.

[CR32] Xu G, Shi H, Ren L, Gou H, Gong D, Gao X, Huang N (2015). Enhancing the anti-colon cancer activity of quercetin by self-assembled micelles. Int J Nanomed.

[CR33] Yao RR, Li JH, Zhang R, Chen RX, Wang YH (2018). M2-polarized tumor-associated macrophages facilitated migration and epithelial-mesenchymal transition of HCC cells via the TLR4/STAT3 signaling pathway. World J Surg Oncol.

[CR34] Zandi Z, Kashani B, Poursani EM, Bashash D, Kabuli M, Momeny M, Mousavi-Pak SH, Sheikhsaran F, Alimoghaddam K, Mousavi SA, Ghaffari SH (2019). TLR4 blockade using TAK-242 suppresses ovarian and breast cancer cells invasion through the inhibition of extracellular matrix degradation and epithelial-mesenchymal transition. Eur J Pharmacol.

[CR35] Zhang Y, Wei C, Guo CC, Bi RX, Xie J, Guan DH, Yang CH, Jiang YH (2017). Prognostic value of microRNAs in hepatocellular carcinoma: a meta-analysis. Oncotarget.

[CR36] Zhong X, Zhang L, Li Y, Li P, Li J, Cheng G (2018). Kaempferol alleviates ox-LDL-induced apoptosis by up-regulation of miR-26a-5p via inhibiting TLR4/NF-kappaB pathway in human endothelial cells. Biomed Pharmacother.

[CR37] Zhou W, Chen X, Hu Q, Chen X, Chen Y, Huang L (2018). Galectin-3 activates TLR4/NF-kappaB signaling to promote lung adenocarcinoma cell proliferation through activating lncRNA-NEAT1 expression. BMC Cancer.

[CR38] Zhou S, Du R, Wang Z, Shen W, Gao R, Jiang S, Fang Y, Shi Y, Chang A, Liu L, Liu C, Li N (2019). TLR4 increases the stemness and is highly expressed in relapsed human hepatocellular carcinoma. Cancer Med.

